# Cost of maternal near miss and potentially life-threatening conditions, Kenya

**DOI:** 10.2471/BLT.20.283861

**Published:** 2021-09-28

**Authors:** Kenneth Juma, Joshua Amo-Adjei, Taylor Riley, Winstoun Muga, Michael Mutua, Onikepe Owolabi, Martin Bangha

**Affiliations:** a African Population and Health Research Center, P.O. Box 10787, Manga Cl, Nairobi, Kenya.; b Department of Population and Health, University of Cape Coast, Cape Coast, Ghana.; c Guttmacher Institute, New York, New York, United States of America.

## Abstract

**Objective:**

To estimate the direct costs of treating women with maternal near misses and potentially life-threatening conditions in Kenya and the factors associated with catastrophic health expenditure for these women and their households.

**Methods:**

As part of a prospective, nationally representative study of all women with near misses during pregnancy and childbirth or within 42 days of delivery or termination of pregnancy, we compared the cost of treating maternal near-miss cases admitted to referral facilities with that of women with potentially life-threatening conditions. We used logistic regression analysis to assess clinical, demographic and household factors associated with catastrophic health expenditure.

**Findings:**

Of 3025 women, 1180 (39.0%) had maternal near misses and 1845 (61.0%) had potentially life-threatening conditions. The median cost of treating maternal near misses was 7135 Kenyan shillings (71 United States dollars, US$) compared with 2690 Kenyan shillings (US$ 27) for potentially life-threatening conditions. Of the women who made out-of-pocket payments, 26.4% (122/462) experienced catastrophic expenditure. The highest median costs for treatment of near misses were in Nairobi and Central region (22 220 Kenyan shillings; US$ 222). Women with ectopic pregnancy complications and pregnancy-related infections had the highest median costs of treatment, at 7800 Kenyan shillings (US$ 78) and 3000 Kenyan shillings (US$ 30), respectively. Pregnancy-related infections, abortion, ectopic pregnancy, and treatment in secondary and tertiary facilities were significantly associated with catastrophic expenditure.

**Conclusion:**

The cost of treating maternal near misses is high and leads to catastrophic spending through out-of-pocket payments. Universal health coverage needs to be expanded to guarantee financial protection for vulnerable women.

## Résumé

**Objectif:**

Estimer les coûts directs engendrés par la prise en charge des mères ayant évité un décès de justesse et par les maladies potentiellement mortelles au Kenya, ainsi que les facteurs liés aux dépenses de santé catastrophiques pour ces femmes et leur foyer.

**Méthodes:**

Une étude prospective et représentative à l'échelle nationale a été menée auprès de l'ensemble des femmes ayant évité un décès de justesse durant la grossesse et l'accouchement, ou dans les 42 jours suivant l'accouchement ou l'interruption de grossesse. C'est dans ce contexte que nous avons comparé le coût du traitement des décès maternels évités de justesse avec celui des femmes souffrant de maladies potentiellement mortelles. Nous avons procédé à une analyse de régression logistique afin d'évaluer les facteurs cliniques, démographiques et domestiques liés aux dépenses de santé catastrophiques.

**Résultats:**

Sur un total de 3025 femmes, 1180 (39,0%) avaient évité un décès de justesse et 1845 (61,0%) présentaient des maladies potentiellement mortelles. La prise en charge des décès maternels évités de justesse coûtait en moyenne 7135 shillings kényans (71 dollars américains), tandis que les maladies potentiellement mortelles revenaient à 2690 shillings kényans (27 dollars américains). Parmi les femmes contraintes de payer elles-mêmes ces frais, 26,4% (122/462) ont fait face à des dépenses catastrophiques. C'est à Nairobi et dans la Province centrale que la prise en charge des décès évités de justesse s'est avérée la plus onéreuse (22 220 shillings kényans; 222 dollars américains). Les coûts moyens les plus élevés étaient assumés par les femmes ayant subi des complications à cause d'une grossesse ectopique (7800 shillings kényans; 78 dollars américains) et des infections liées à la grossesse (3000 shillings kényans; 30 dollars américains). Les infections liées à la grossesse, l'avortement, les grossesses ectopiques et les traitements dans des établissements secondaires et tertiaires étaient fréquemment synonymes de dépenses catastrophiques.

**Conclusion:**

Le montant que représente la prise en charge des décès maternels évités de justesse est considérable et entraîne des dépenses catastrophiques lorsqu'il doit être déboursé par la patiente elle-même. Il est impératif d'étendre la couverture maladie universelle afin d'assurer la protection financière des femmes vulnérables.

## Resumen

**Objetivo:**

Estimar los costes directos del tratamiento de las mujeres con morbilidad materna extrema y con enfermedades potencialmente mortales en Kenia, así como los factores asociados a los gastos sanitarios catastróficos para estas mujeres y sus hogares.

**Métodos:**

Como parte de un estudio de cohortes y representativo a nivel nacional de todas las mujeres con morbilidad extrema durante el embarazo y el parto o dentro de los 42 días posteriores al parto o a la interrupción del embarazo, se comparó el coste del tratamiento de los casos de morbilidad extrema ingresados en centros de referencia con el de las mujeres con enfermedades potencialmente mortales. Se utilizó un análisis de regresión logística para evaluar los factores clínicos, demográficos y domésticos asociados a los gastos sanitarios catastróficos.

**Resultados:**

De 3025 mujeres, 1180 (39,0 %) tuvieron morbilidad materna extrema y 1845 (61,0 %) padecieron enfermedades potencialmente mortales. El coste medio del tratamiento de los casos de morbilidad materna extrema fue de 7135 chelines kenianos (71 dólares estadounidenses, USD), en comparación con los 2690 chelines kenianos (27 USD) de las enfermedades potencialmente mortales. De las mujeres que pagaron de su bolsillo, el 26,4 % (122/462) tuvo gastos catastróficos. La mediana de los costes más elevados para el tratamiento de la morbilidad materna extrema se registró en Nairobi y en la región central (22 220 chelines kenianos; 222 USD). Las mujeres con problemas de embarazo ectópico y con infecciones causadas por el embarazo tuvieron los costes medios de tratamiento más elevados, 7800 chelines kenianos (78 USD) y 3000 chelines kenianos (30 USD), respectivamente. Las infecciones causadas por el embarazo, el aborto, el embarazo ectópico y el tratamiento en centros de atención secundaria y terciaria se asociaron de manera significativa con los gastos catastróficos.

**Conclusión:**

El coste del tratamiento de la morbilidad materna extrema es alto y genera un gasto catastrófico mediante los pagos de bolsillo. Se debe ampliar la cobertura sanitaria universal para garantizar la protección financiera de las mujeres vulnerables.

## ملخص

**الغرض:**

تقدير التكاليف المباشرة لمعالجة النساء اللاتي كن على وشك الوفاة أثناء الولادة، والظروف المحتمل تهديدها للحياة في كينيا، والعوامل المرتبطة بالإنفاق الصحي الكارثي لهؤلاء النساء وأسرهن.

**الطريقة:**

كجزء من دراسة مستقبلية تمثيلية على المستوى الوطني لجميع النساء اللاتي كن على وشك الوفاة أثناء الحمل والولادة، أو في غضون 42 يومًا من الولادة أو نهاية الحمل، قمنا بمقارنة تكلفة معالجة حالات الأمهات على وشك الوفاة، والتي تم قبولها في مرافق الإحالة مع تكلفة النساء اللاتي تعرضن لظروف قد تهدد الحياة. لقد استخدمنا تحليل الانحدار اللوجستي لتقييم العوامل السريرية والديموغرافية والأسرية المرتبطة بالإنفاق الصحي الكارثي.

**الاستنتاج:**

من بين 3025 امرأةً، كانت 1180 (39.0%) منهن على وشك الوفاة، وتعرضت 1845 (61.0%) منهم لظروف قد تهدد الحياة. كان متوسط تكلفة علاج الأمهات اللاتي كن على وشك الوفاة 7135 شلنًا كينيًا (71 دولارًا أمريكيًا)، مقارنة بـ 2690 شلنًا كينيًا (27 دولارًا أمريكيًا) للحالات التي قد تهدد الحياة. من بين النساء اللاتي سددن المدفوعات من أموالهن، تعرضت 26.4% (122/462) منهن لنفقات كارثية. وكان أعلى متوسط لتكاليف علاج الحالات التي كانت على وشك الوفاة في نيروبي والمنطقة الوسطى (2220 شلنًا كينيًا؛ 222 دولارًا أمريكيًا). كانت النساء المصابات بمضاعفات الحمل خارج الرحم وحالات العدوى المرتبطة بالحمل، هن أصحاب أعلى متوسط لتكاليف العلاج، 7800 شلن كيني (78 دولارًا أمريكيًا) و3000 شلن كيني (30 دولارًا أمريكيًا)، على التوالي. ارتبطت حالات العدوى المرتبطة بالحمل والإجهاض والحمل خارج الرحم والمعالجة في المرافق الثانوية والثالثية بشكل كبير بالنفقات الكارثية.

**الاستنتاج:**

تكلفة علاج حالات الأمهات الوشيكة على الوفاة مرتفعة، وتؤدي إلى إنفاق كارثي من خلال المدفوعات الشخصية. تحتاج التغطية الصحية الشاملة للتوسيع لضمان الحماية المالية للنساء المعرضات للخطر.

## 摘要

**目的:**

估计治疗肯尼亚女性的孕产妇险兆事件和可能危及生命的情况的直接成本，以及导致这些女性及其家庭将这笔成本视为灾难性医疗支出的影响因素。

**方法:**

作为一项具有前瞻性和全国代表性研究的一部分，我们研究了所有在怀孕和分娩期间、分娩或终止妊娠 42 天内发生险兆事件的女性，比较了孕产妇险兆事件（包括转诊病例）的治疗费用和可能危及女性生命的情况的治疗费用。我们使用逻辑回归分析来评估导致这笔费用成为灾难性医疗支出的临床、人口统计和家庭因素。

**结果:**

在 3025 名女性中，1180 (39.0%) 名女性经历过孕产妇险兆事件，1845 (61.0%) 名女性经历过可能危及生命的情况。孕产妇险兆事件的治疗费用中位数为 7135 肯尼亚先令（71 美元），而可能危及生命的治疗费用中位数成本则为 2690 肯尼亚先令（27 美元）。在自付费用的女性中，这笔费用对于 26.4%（122/462）的女性来说是灾难性医疗支出。内罗比和中部地区的险兆事件的治疗费用中位数最高（22,220 肯尼亚先令，即 222 美元）患有异位妊娠并发症和妊娠相关感染的女性的治疗费用中位数最高，分别为 7800 肯尼亚先令（78 美元）和 3000 肯尼亚先令（30 美元）。灾难性医疗支出主要与妊娠相关感染、流产、异位妊娠以及在二级和三级机构接受治疗有关。

**结论:**

治疗孕产妇险兆事件的费用很高，所以对于需要自付费用的患者及其家庭而言是一笔灾难性支出。因此，需要扩大全民医疗保险覆盖范围，以确保弱势女性获得财务保障。

## Резюме

**Цель:**

Оценить прямые затраты на лечение женщин с осложнениями во время родов, представляющими угрозу для жизни, и с потенциально опасными для жизни состояниями в Кении, а также факторы, связанные с катастрофическими расходами на медицинское обслуживание таких женщин и их домохозяйств.

**Методы:**

В рамках проспективного, репрезентативного на национальном уровне исследования с участием женщин с осложнениями, представляющими угрозу для жизни во время беременности и родов или в течение 42 дней после родов или прерывания беременности, авторы сравнили затраты на лечение случаев осложнения, представляющих угрозу для жизни женщин, поступивших в специализированные медицинские учреждения, с затратами на лечение женщин с потенциально опасными для их жизни состояниями. Авторы использовали логистический регрессионный анализ для оценки клинических, демографических и бытовых факторов, связанных с катастрофическими расходами на медицинское обслуживание.

**Результаты:**

Из 3025 женщин 1180 (39,0%) имели осложнения, представляющие угрозу для их жизни, а 1845 женщин (61,0%) имели потенциально опасные для их жизни состояния. Средняя стоимость лечения осложнений, представляющих угрозу для жизни, составила 7135 кенийских шиллингов (71 доллар США) по сравнению с 2690 кенийскими шиллингами (27 долларов США) при лечении потенциально опасных для жизни состояний. 26,4% (122/462) женщин, которые оплачивали услуги из собственных средств, понесли катастрофические расходы. Самые высокие средние затраты на лечение осложнений, представляющих угрозу для жизни, отмечены в Найроби и Центральном регионе (22 220 кенийских шиллингов (222 доллара США)). Женщины с осложнениями внематочной беременности и со связанными с беременностью инфекциями имели самые высокие средние затраты на лечение: 7800 кенийских шиллингов (78 долларов США) и 3000 кенийских шиллингов (30 долларов США) соответственно. Инфекции, связанные с беременностью, аборты, внематочная беременность и лечение в учреждениях вторичного и третичного уровня были в значительной степени связаны с катастрофическими расходами.

**Вывод:**

Стоимость лечения осложнений, представляющих угрозу для жизни, достаточно высока и приводит к катастрофическим расходам из-за оплаты услуг из собственных средств. Необходимо расширить всеобщий охват услугами здравоохранения, чтобы гарантировать финансовую защиту уязвимых групп женщин.

## Introduction

Many countries in sub-Saharan Africa have increasingly committed to tackling maternal illnesses and deaths through a mix of interventions. For instance, in the past 15 years, the Kenyan government has implemented structural reforms mainly focused on increasing access to and reducing the cost of care for women. These reforms include the 2006 care subsidy voucher programme,^1^ the 2013 free maternity care programme in public facilities,^2^ which significantly increased facility-based births,^3^ and the health insurance subsidy programme for poor people.^4^ Yet, preventable maternal deaths are still high in Kenya, where the maternal mortality ratio is estimated at 510 maternal deaths per 100 000 live births.^5^

The spectrum of maternal morbidity as described by the World Health Organization (WHO) ranges from mild to moderate, severe (potentially life-threatening conditions), maternal near miss or death. WHO defines maternal near miss as a woman who nearly died but survived a complication that occurred during pregnancy or within 42 days of delivery or termination.^6^ On the other hand, potentially life-threatening conditions are severe complications that may progress to near misses but may also resolve with clinical care, are not at the end of the spectrum of morbidity, and typically require less intensive care and resources to manage than maternal near miss. 

Maternal mortality ratio is a popular indicator for assessing progress in maternal health, but it is not adequate to understand the full scale of maternal health-care provision and outcomes,^7^ because maternal deaths are infrequent events.^8^ Thus, maternal near-miss events, which occur 5–10 times more frequently than maternal deaths and often require intensive care, attendance by highly skilled staff and extended hospital stays,^9^^–^^16^ may be used as a proxy to explore the circumstances surrounding maternal deaths. 

In low- and middle-income countries where health systems may be weak and there is little financial protection for vulnerable people, experiencing severe obstetric complications such as maternal near miss likely increases the risk of catastrophic expenditure for women and their households compared with less severe complications or an uncomplicated delivery. Catastrophic expenditure is any cost incurred in the process of seeking health care that threatens a household’s ability to meet its subsistence needs,^17^ and could push households into financial hardship and poverty.^16^ At the core of the universal health coverage (UHC) target of the sustainable development goals (SDGs) is guaranteeing financial protection for all people seeking health care and expanding services. Despite increased investment in UHC efforts in many low- and middle-income countries, recent data suggest that progress towards financial protection and service coverage has been limited.^18^

In Kenya, the elimination of user fees for maternity care should theoretically cover antenatal care, deliveries, postnatal care, referrals and family planning. In practice however, this only covers deliveries.^19^ Kenyan health-care financing is predominantly through social health insurance and non-contributory mechanisms (government tax and donors). The National Health Insurance Fund currently covers about 18% of Kenyans, while private insurance covers only about 1% of the population. Most Kenyans depend on out-of-pocket payments. Whereas maternity care is free, patients must often make other advance or post-service payments in cases of emergency. Occasionally, patients who are unable to pay rely on social networks (e.g. churches and friends) for support or, in extreme cases, health facilities may exempt or waive hospital bills. These out-of-pocket payments are a significant financial barrier to accessing emergency obstetric care and result in delays, which may lead to clinical deterioration and increase women’s risk of experiencing a maternal near miss and facing catastrophic expenditure.^20^^–^^23^

Tackling direct and indirect financial expenditure in accessing health care for severe obstetric complications is thus essential to reducing the maternal mortality ratio.^24^^–^^27^ Understanding the magnitude of the financial burden associated with maternal near miss and other levels of complications is a starting point for efforts to eliminate financial barriers and guarantee protections against catastrophic expenditure.^27^^–^^30^ However, few studies have been done to understand the economic consequences of pregnancy complications, how costs increase with the severity of morbidity and the likely impact on women and their households. This information is important to better understand the financial implications of varying levels of severe obstetric complications on households in Kenya and to enable the government to adapt financing and payment mechanisms to provide adequate financial protection for all women. Therefore, we assessed the total financial costs to women and/or their families associated with maternal near-miss events in Kenya and compared these costs to the costs of potentially life-threatening conditions. We also examined the factors associated with incurring catastrophic expenditure among women making out-of-pocket payments for their care.

## Methods

### Study design and sample

This study is part of a larger prospective nationally representative study conducted from February to May 2018 in Kenya that estimated the incidence of maternal near miss and the quality of clinical management.^31^ The larger study was interested in capturing all cases of maternal near miss; as such, our inclusion criteria captured all women admitted to referral-level facilities (i.e. level IV, V and VI facilities) in Kenya who developed or received an intervention for a potentially life-threatening condition resulting from pregnancy, childbirth or within 42 days of delivery or termination of pregnancy. We also included women who had presented in the facility as a maternal near miss or had died of pregnancy-related causes. Using the WHO definition of maternal near miss,^6^ we then applied clinical algorithms to determine which women had experienced a maternal near-miss event out of all the eligible women in the study.

### Sampling and recruitment

The Kenyan public health system has six levels of health care as defined in the 2014 Kenya Health Sector Strategic and Investment Plan: level I community units; level II dispensaries; level III health centres; level IV primary referral facilities; level V secondary referral facilities; and level VI tertiary referral facilities.^32^ Since maternal near-miss events are severe complications that may require surgery, we included all level V (16) and VI (two) facilities, which are designated to perform caesarean sections and are more likely to handle maternal near-miss cases. We then generated a random sample of 46 level IV facilities from a total of 426 (stratified by region), which are the primary treatment and referral facilities for severe pregnancy-related complications in areas where there are no immediate higher-level facilities. Within the study facilities, we recruited into the study all women admitted with potentially life-threatening conditions or those who developed these conditions during their hospital stay.

### Data collection

Each facility had at least one trained clinician, who was a doctor, clinical officer or nurse. In bigger facilities with higher caseloads, two or three trained clinicians assessed patients admitted with obstetric emergencies for eligibility for the study. If a patient was eligible, and in a stable condition, the interviewer requested informed consent and administered the clinical study questionnaire.^31^ We linked all women who consented to participate in the cost component of the study to trained fieldworkers at discharge who interviewed them or their caretakers. Data collected included both direct medical costs (e.g. medicines, consultations or surgeries and diagnostic procedures) and direct non-medical costs (e.g. transport, food and accommodation) incurred during the time of the maternal near-miss event. We did not measure indirect costs (e.g. lost earnings and waiting time) and intangible costs (e.g. pain, inconvenience and anxiety). Interviewers used a tablet-based questionnaire to record data on SurveyCTO and then uploaded the data to a central server based at the African Population and Health Research Center. Interviewers conducted the interviews in a private location within the health facilities, determined in consultation with facility management. 

### Data analysis

We used STATA, version 15 (StataCorp LLC, College Station, United States of America) for statistical analysis. We only included patients with both clinical and financial data in the analysis. We conducted exploratory analyses to describe the patient characteristics and estimated cost of treatment. Given the skewed distribution of the cost data, we estimated the direct cost of care using medians and interquartile ranges (IQR). In addition, we used the Mann–Whitney U test to compare the cost of treatment for maternal near-miss events and potentially life-threatening conditions. We summarized sociodemographic characteristics and underlying clinical complications of the participants as proportions.

### Catastrophic expenditure

We categorized health spending as catastrophic when out-of-pocket health expenditure exceeded a certain proportion of total household consumption. Using just one threshold could result in misinterpretation of important factors. Therefore, we used two previously applied consumption thresholds (10% of total expenditure and 40% of non-food expenditure)^28^^–^^30^ to women who made out-of-pocket payments (available in the data repository).^33^ We applied both thresholds as a sensitivity check and to determine which threshold yielded stronger explanation for our regression model (available in the data repository).^33^ The frequency of catastrophic spending was the proportion of households that exceeded either of these two thresholds. To assess the clinical, demographic and household characteristics associated with catastrophic expenditures, we used multiple logistic regression analysis with the outcome dichotomized as: did or did not experience catastrophic expenditure. We included the following covariates in the final model: residence, level of education, length of hospital stay, level of treatment facility and underlying cause of severe obstetric complications. We report odds ratios (OR) and corresponding 95% confidence intervals (CI).

### Ethical approval

The Kenya Medical Research Institute Scientific Ethics Review Unit approved the study, as did the Guttmacher Institute institutional review board and the National Commission for Science, Technology and Innovation, Kenya.

## Results

### Characteristics

A total of 3082 women (weighted for level of health facility and region) were eligible for inclusion in the study and 3025 participated in the cost component (response rate of 98.2%). The other 57 women either declined to participate, were discharged before interviews, were referred to other facilities with higher or same levels of care, or died. Of the 3025 women in the study, 1180 (39.0%) had had a maternal near miss and 1845 (61.0%) had had potentially life-threatening conditions. About half the respondents (51.1%; 1545/3025) were aged 20–29 years (Table 1). Most women had primary or secondary level education (74.5%; 2255/3025), were unemployed (63.0%; 1907/3025) and lived in rural areas (69.6%; 2105/3025). Of the women who had experienced a maternal near-miss event, 57.2% (675/1180) were between 20 and 29 years and 65.6% (774/1180) lived in rural areas.

**Table 1:** Sociodemographic characteristics of women experiencing severe obstetric complications, Kenya, 2018

Characteristic	No. (%)		
	Maternal near miss (*n* = 1180)	Potentially life-threatening conditions (*n* = 1845)	All cases (*n* = 3025)
**Age group (years)**			
15–19	92 (7.8)	236 (12.8)	328 (10.8)
20–24	353 (29.9)	423 (22.9)	776 (25.7)
25–29	322 (27.3)	447 (24.2)	769 (25.4)
30–34	240 (20.3)	441 (23.9)	681 (22.5)
> 35	173 (14.7)	298 (16.2)	471 (15.6)
**Education level^a^**			
Primary	387 (32.8)	721 (39.1)	1108 (36.6)
Secondary	467 (39.6)	680 (36.9)	1147 (37.9)
Tertiary	181 (15.4)	318 (17.2)	499 (16.5)
No education	144 (12.2)	126 (6.8)	270 (8.9)
**Occupation**			
Employed	444 (37.6)	674 (36.5)	1118 (37.0)
Unemployed	736 (62.4)	1171 (63.5)	1907 (63.0)
**Residence**			
Rural	774 (65.6)	1331 (72.1)	2105 (69.6)
Urban	406 (34.4)	514 (27.9)	920 (30.4)
**Religion**			
Catholic	373 (31.6)	382 (20.7)	755 (25.0)
Protestant	695 (58.9)	1249 (67.7)	1944 (64.3)
Muslim	112 (9.5)	214 (11.6)	326 (10.8)
**Marital status**			
Married	882 (74.7)	1463 (79.3)	2345 (77.5)
Divorced or widowed	58 (4.9)	88 (4.8)	146 (4.8)
Never married	240 (20.3)	294 (15.9)	534 (17.7)
**Region**			
Coast and North Eastern	112 (9.5)	196 (10.6)	308 (10.2)
Eastern	82 (6.9)	114 (6.2)	196 (6.5)
Nairobi and Central	300 (25.4)	168 (9.1)	468 (15.5)
Nyanza and Western	302 (25.6)	1097 (59.5)	1399 (46.2)
Rift Valley	384 (32.5)	270 (14.6)	654 (21.6)

^a^ Missing data for one observation in the maternal near-miss group.

Notes: All estimates are weighted from the 3-month study period and include only patients who consented to be interviewed for both the cost and clinical surveys. Inconsistencies arise in some values due to rounding.

### Total direct costs 

The median total cost of treatment for a maternal near-miss event in Kenyan shillings (1 United States dollar = 100 Kenyan shillings in May 2018) was 7135 Kenyan shillings (IQR: 50–271 068) compared with 2690 Kenyan shillings (IQR: 50–68 293) for potentially life-threatening conditions. A significant share of the total cost of treating maternal near misses was attributed to direct medical costs (4000  Kenyan shillings; IQR: 100–161 434), e.g. for medicines, laboratory tests and X-rays, compared with non-medical costs (1600 Kenyan shillings; IQR: 50–40 900), e.g. for transport and food (Table 2). The difference between median costs of maternal near-miss cases and potentially life-threatening conditions was significant across all categories of medical (*P* < 0.001) and non-medical costs (*P* = 0.001).

**Table 2:** Cost of severe obstetric complications by type of cost, Kenya, 2018

Type of cost	Women, no.	Range of costs, Kenyan shillings	Median cost, Kenyan shillings (IQR)	*P* ^a^
**Direct medical**				< 0.001
Maternal near miss	246	20–322 295	4000 (100–161 434)	
Potentially life-threatening conditions	191	10–127 734	2700 (50–49 500)	
All cases	437	10–322 959	3367 (50–161 434)	
**Non-medical **				0.001
Maternal near miss	311	30–177 250	1600 (50–40 900)	
Potentially life-threatening conditions	270	20–41 200	800 (50–18 350)	
All cases	581	20–177 250	1000 (30–41 200)	
**Total**				< 0.001
Maternal near miss	328	30–521 800	7135 (50–271 068)	
Potentially life-threatening conditions	281	50–136 284	2690 (50–68 293)	
All cases	609	30–521 800	5200 (50–271 068)	

IQR: interquartile range.

^a^ The Mann–Whitney U test was used to compare the costs for the maternal near-miss cases and cases with potentially life-threatening conditions under the null hypothesis that no difference exists in the distribution.

Notes: All estimates are weighted from the 3-month study period and include only patients who consented to be interviewed for both the cost and clinical surveys. 1 United States dollar = 100 Kenyan shillings in May 2018.

### Regional variations in cost

**Fig. 1:**
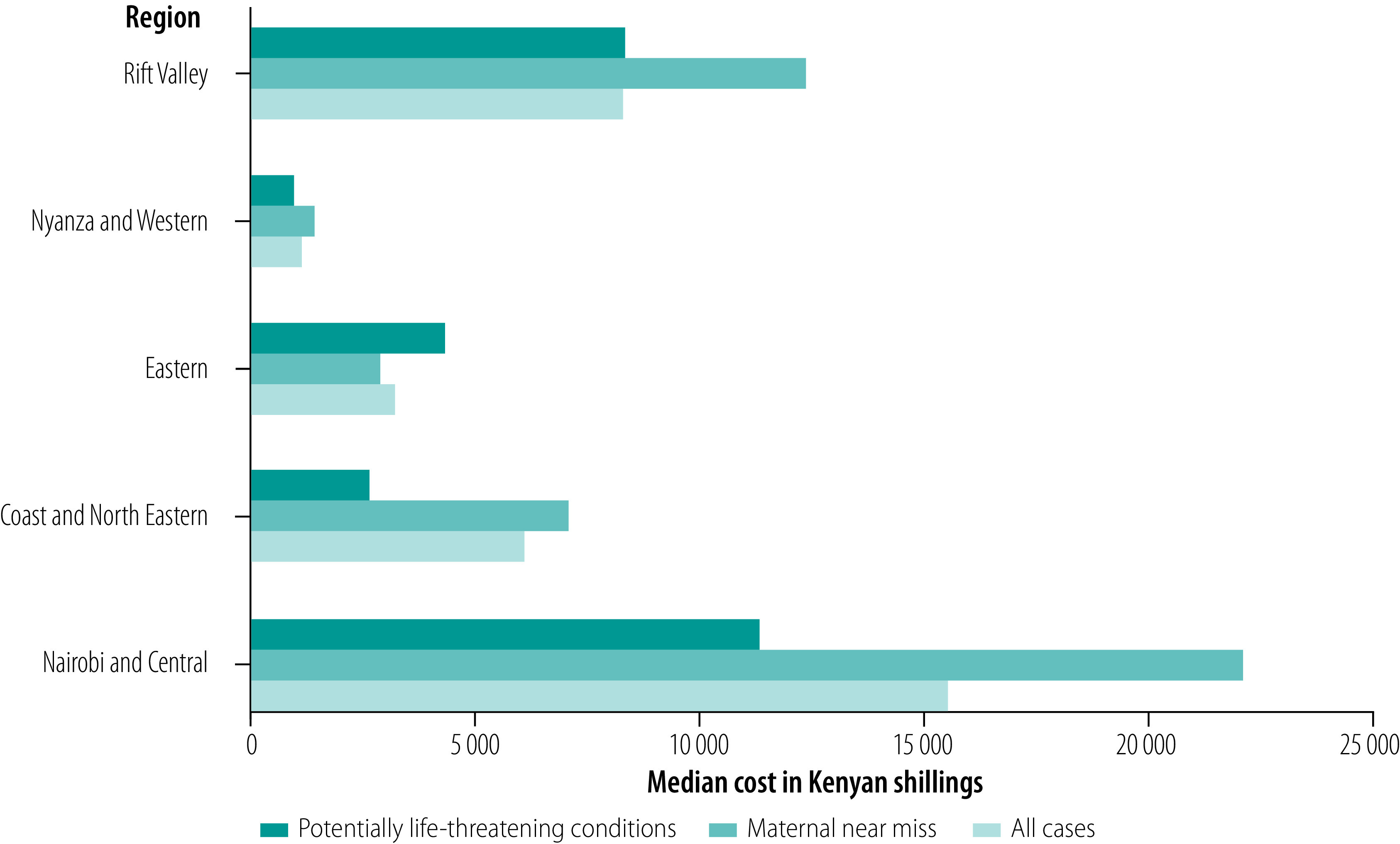
Direct costs of severe obstetric complications by region, Kenya, 2018

Note: 1 United States dollar = 100 Kenyan shillings in May 2018.

The median cost of treating near-miss episodes varied considerably by region, with the highest costs in Nairobi and Central region (22 220 Kenyan shillings; IQR: 570–135 612) and Rift Valley region (12 395 Kenyan shillings; IQR: 500–235 133), and the lowest in Nyanza and Western region (1400 Kenyan shillings; IQR: 50–38 890). Across all regions, the median costs of treatment for maternal near-miss patients were higher than for patients with potentially life-threatening conditions, except in the Eastern region (Fig. 1).

### Cost by underlying complication

Disaggregating costs by type of complication, the highest median costs were for women with ectopic pregnancy complications (7800 Kenyan shillings; IQR: 400–48 932), pregnancy-related infections (3000 shillings; IQR: 400–167 434) and medical, surgical or neurological complications (2600 Kenyan shillings; IQR: 200–17 240). The lowest median costs were for women with hypertensive disorders (1400 Kenyan shillings; IQR: 50–194 175) and obstetric haemorrhage (1500 Kenyan shillings; IQR: 50–120 916; Table 3).

**Table 3:** Cost of treatment for severe obstetric complications by underlying cause, Kenya, 2018

Cause of complication	Women, no.	Range of costs, Kenyan shillings	Median cost, Kenyan shillings (IQR)
Hypertensive disorders	643	50–521 800	1400 (50–194 175)
Obstetric haemorrhage	1709	30–167 434	1500 (50–120 916)
Pregnancy-related infections	179	50–306 829	3000 (400–167 434)
Other obstetric complications^a^	658	50–136 284	1500 (50–76 000)
Severe anaemia	426	60–306 829	2000 (100–120 916)
Pregnancy with abortive outcome	370	50–235 133	2240 (660–78 045)
Ectopic pregnancy	434	200–99 365	7800 (400–48 932)
Medical, surgical or neurological disease or complications^b^	20	200–193 762	2600 (200–17 240)

IQR: interquartile range.

^a^ Other obstetric disease: obstructed labour complications, placenta complications, uterus complications, stillbirth complications, preterm complications.

^b^ Cardiovascular complications, cerebrovascular accident, ascites complications, pulmonary complications, renal and blood complications.

Notes: All estimates are weighted from the 3-month study period and include only patients who consented to be interviewed for both the cost and clinical surveys. 1 United States dollar = 100 Kenyan shillings in May 2018.

### Source of financing

Just over half of maternal near-miss patients (56.4%; 665/1180) and 65.0% (1199/1845) of patients with potentially life-threatening conditions made out-of-pocket payments for treatment. Only 26.0% (307/1180) of near-miss patients had some form of insurance cover, including the National Hospital Insurance Fund, Community Health Insurance Scheme or private health insurance. One third of near-miss patients (30.0%; 354/1180) had their medical bills waived or they were exempted from paying, and 7.0% (83/1180) had other ways of paying for their medical bills (e.g. paid by friends, allowed to pay later).

### Catastrophic expenditure

**Fig. 2:**
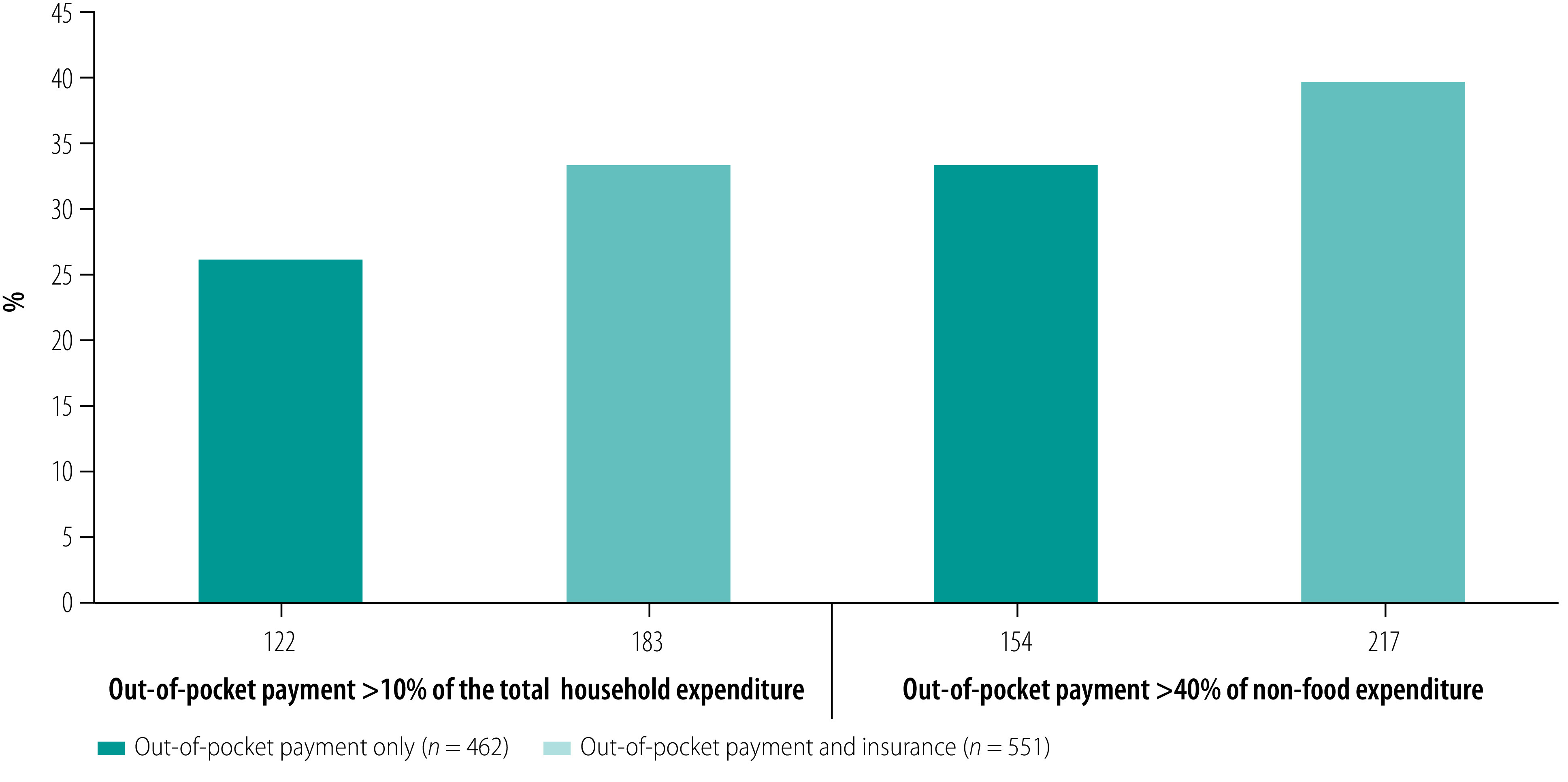
Proportion of households with catastrophic expenditure after making out-of-pocket payments for severe obstetric complications, Kenya, 2018

Based on the threshold of health expenditure of over 40% of non-food expenditures, more than one in three (33.3%; 154/462) households paying entirely through out-of-pocket payments experienced catastrophic expenditure. Similarly, more than a quarter of such households (26.4%; 122/462) incurred catastrophic expenditures using the threshold of over 10% of total household expenditure (Fig. 2). Proportions of patients who experienced catastrophic expenditures increased across the two thresholds when we included those who made payments both out of pocket and through insurance cover.

### Factors associated with catastrophic expenditure 

In a logistic regression analysis to examine the socioeconomic and clinical factors associated with catastrophic expenditure at the two thresholds, both models significantly explained the observed variances in catastrophic expenditure. The 40% threshold gave a better explanation of the variances: with the 10% threshold, the model summary was *χ^2^* = 91.539, *P* < 0.0005 and Nagelkerke *R*^2^ = 26.2%; and with the 40% threshold, the model summary was *χ^2^* = 117.388, *P* < 0.0005 and Nagelkerke *R*^2^ = 31.2%. Using the over 10% of total expenditure threshold, the likelihood of catastrophic spending for women with pregnancy-related infections was 3.1 times greater than for women without infections, 3.7 times greater for women with abortion complications and 2.2 times greater for women with ectopic pregnancy complications (Table 4). Furthermore, the likelihood of catastrophic spending was 6.5 times greater for women in level VI than level IV hospitals and 1.9 times greater for women in level V than level IV hospitals. Rural residence was also associated with catastrophic expenditure (OR: 1.94; 95% CI: 1.25–2.99).

**Table 4:** Factors associated with catastrophic expenditure among women who made out-of-pocket payments for severe obstetric complications, Kenya, 2018

Characteristic	aOR (95% CI)	
	Over 10% of total expenditure	Over 40% of non-food expenditure
**Area of residence**		
Urban	Ref.	Ref.
Rural	1.935 (1.25–2.99)	2.670 (1.75–4.09)
**Education level**		
No education	Ref.	Ref.
Primary	0.37 (0.18–0.78)	0.39 (0.19–0.81)
Secondary	0.36 (0.17–0.76)	0.26 (0.12–0.54)
Tertiary	0.64 (0.28–1.50)	0.42 (0.19–0.97)
**Underlying cause of severe obstetric complication^a^**		
Pregnancy-related infections	3.06 (1.52–6.20)	3.15 (1.55–6.41)
Pregnancy with abortive outcome	3.73 (2.00–6.94)	4.21 (2.27–7.79)
Ectopic pregnancy complications	2.21 (1.24–3.93)	2.35 (1.35–4.08)
**Inpatient days**	1.04 (1.02–1.06)	1.03 (1.01–1.05)
**Hospital level**		
IV	Ref.	Ref.
V	1.85 (1.03–3.32)	2.51 (1.46–4.33)
VI	6.53 (3.54–12.04)	6.30 (3.48–11.43)
**Constant^b^**	0.14	0.17

aOR: adjusted odds ratio; CI: confidence interval; Ref.: reference category.

^a^ Reference categories are women who did not have the complications.

^b^ Constant/intercept coefficient: this is the expected value of the log-odds of the outcome variable (in this case catastrophic expenditure) when all of the predictor variables equal zero.

Note: We only included patients who only made out-of-pocket payments in this analysis. We excluded patients who paid through insurance or those who combined out-of-pocket and insurance payments.

Using the over 40% of non-food expenditure threshold, having pregnancy-related infections was associated with 3.1 times higher odds of making catastrophic payments, while the odds for women with abortion complications were 4.2 times higher, and the odds for women with ectopic pregnancy complications were 2.3 times higher. In addition, the odds of catastrophic expenditure was 6.3 times higher for women in level VI hospitals and 2.5 times higher for women in level V hospitals compared with women in level IV facilities (Table 4).

## Discussion

Generally, the direct medical cost (i.e. for consultation, medicines and laboratory procedures) was higher for treating maternal near misses than treating potentially life-threatening conditions. Our findings illustrate wide variability in the cost of treating maternal near misses in the different regions of Kenya, with Nairobi and Central regions recording the highest median cost, while Nyanza and Western regions reported the lowest median cost.

Our findings suggest that the financial burden arising from maternal near-miss events in Kenya is high. The median cost of treating an episode of maternal near miss was 7135 Kenyan shillings, which is expensive given that the average monthly wage in Kenya was 6900 Kenyan shillings in 2018.^34^ Most of the maternal near-miss patients made out-of-pocket payments for their medical care, and more than a quarter of patients with severe obstetric complications who made out-of-pocket payments suffered catastrophic expenditure. Households that experience catastrophic expenditure have been found unable to meet subsistence needs such as rent and food and may be forced to turn to loans and sometimes liquidation of household assets.^22^^,^^28^ Furthermore, catastrophic expenditure was associated with certain severe obstetric complications, rural residence and attending level V and VI hospitals. These findings reflect the treatment needs for such complications, for example, treating infections that may require costly drug therapies, specialized staff and longer admissions.^28^

The estimated cost of treating maternal near misses in our study was considerably higher than reported in Ghana for treatment of all pregnancy-related complications, where the median expenditure by households per complication was US$ 32.03.^35^ These differences may be due to the fact that our study focused on more severe maternal complications (maternal near-miss events), which may require longer hospital stays, sophisticated treatment procedures and attendance by highly specialized health staff, and hence result in higher costs of care. On the other hand, the Ghana study focused on all pregnancy-related complications, a significant proportion of which may not have been severe. However, in the context of resource-constrained settings, economically vulnerable women who experience near-miss events risk potential catastrophic expenditure with severe disruptions to household finances in the short and long term. Our findings also highlight the important role played by individuals and households in financing maternal health services in Kenya. Previous studies have indicated that household spending on pregnancy-related complications not only drains household budgets and resources,^17^^,^^26^ but could disrupt their ability to fund subsistence needs, thus driving more vulnerable households to poverty.^30^^,^^35^

While the Kenyan government has made efforts to address the cost of obstetric care (such as elimination of delivery fees),^2^ our study shows that women with near-miss events pay considerable amounts of money for maternal health care, even in public facilities. Such costs may deter women from using emergency obstetric services altogether^20^ or result in delays in accessing services, which can increase the severity of complications.^36^^,^^37^ In addition, most women who experienced a maternal near miss in referral hospitals did not have insurance to cover the costs of their treatment, and had to pay out of pocket. Fee exemption policies may exist to help mitigate costs, but they tend to neglect some critical components of clinical care such as laboratory and ultrasound services that are typically required to diagnose and manage severe conditions.^38^ Our findings suggest that policies aimed at realizing UHC and reaching SDG targets in Kenya should go beyond funding basic maternity care fees to include treatment for women with severe pregnancy-related complications. One attempt to do this in 2018 in Kenya was the piloting of a free maternity scheme called the Linda Mama, which covered antenatal care, postnatal care, delivery and mother and baby complications in both public and private facilities. The Linda Mama initiative has now been scaled up around the country and has resulted in improved accountability to and expanded benefits for women in informal urban settlements and rural areas who are more vulnerable to catastrophic expenditure.^39^ The coronavirus disease 2019 pandemic has adversely affected maternal health services and exposed vulnerable people to economic adversity,^40^^,^^41^ thus, scaling up UHC is important for these disadvantaged populations.

Apart from the limitations reported in our clinical paper,^31^ this study had other limitations. We did not collect indirect costs incurred during treatment, which include loss in productivity during care and the sequelae following a maternal near-miss event. In capturing costs, we focused on costs paid by patients, rather than total costs of care (some paid by insurance firms). We did not ask women when they made the payments (on admittance, during the hospital stay or at discharge). Even so, our study provides best estimates of the direct cost of care for maternal near misses by capturing direct costs of care before arrival at the health facility, during treatment and during referral.

Our findings confirm that women in Kenya who experience maternal near-miss events incur high out-of-pocket costs. The costs of care that are beyond the reach of individuals limit access to emergency obstetric care and could be catastrophic to individuals and households. Efforts are needed to implement UHC and guarantee financial protection to vulnerable people and access to good-quality maternal health care.
